# Normative values for the Foot Posture Index

**DOI:** 10.1186/1757-1146-1-6

**Published:** 2008-07-31

**Authors:** Anthony C Redmond, Yvonne Z Crane, Hylton B Menz

**Affiliations:** 1 Academic Unit of Musculoskeletal Disease, School of Medicine, University of Leeds, Leeds, UK; 2 Musculoskeletal Research Centre, Faculty of Health Sciences, La Trobe University, Bundoora, Victoria, Australia

## Abstract

**Background:**

The Foot Posture Index (FPI) is a validated method for quantifying standing foot posture, and is being used in a variety of clinical settings. There have however, been no normative data available to date for comparison and reference. This study aimed to establish normative FPI reference values.

**Methods:**

Studies reporting FPI data were identified by searching online databases. Nine authors contributed anonymised versions of their original datasets comprising 1648 individual observations. The datasets included information relating to centre, age, gender, pathology (if relevant), FPI scores and body mass index (BMI) where available. FPI total scores were transformed to interval logit scores as per the Rasch model and normal ranges were defined. Comparisons between groups employed t-tests or ANOVA models as appropriate and data were explored descriptively and graphically.

**Results:**

The main analysis based on a normal healthy population (n = 619) confirmed that a slightly pronated foot posture is the normal position at rest (mean back transformed FPI raw score = +4). A 'U' shaped relationship existed for age, with minors and older adults exhibiting significantly higher FPI scores than the general adult population (F = 51.07, *p *< 0.001). There was no difference between the FPI scores of males and females (2.3 versus 2.5; *t *= -1.44, *p *= 0.149). No relationship was found between the FPI and BMI. Systematic differences from the adult normals were confirmed in patients with neurogenic and idiopathic cavus (F = 216.981, *p *< 0.001), indicating some sensitivity of the instrument to detect a posturally pathological population.

**Conclusion:**

A set of population norms for children, adults and older people have been derived from a large sample. Foot posture is related to age and the presence of pathology, but not influenced by gender or BMI. The normative values identified may assist in classifying foot type for the purpose of research and clinical decision making.

## Background

Variations in foot posture are thought to influence the function of the lower limb and may therefore play a role in predisposition to overuse injury [1-4]. Despite these observations, there is still considerable disagreement regarding the most appropriate method for categorizing foot type [5]. A wide array of techniques have been used, including visual observation [3,6], various footprint parameters [7,8], measurement of frontal plane heel position [9,10] and assessment of the position of the navicular tuberosity [11].

Recently, a six-item criterion reference tool (the Foot Posture Index, or FPI) was developed in response to a requirement for a quick, easy and reliable method for measuring foot position in a variety of clinical settings [12]. The FPI consists of six validated, criterion-based observations of the rearfoot and forefoot of a subject standing in a relaxed position. The rearfoot is assessed via palpation of the head of the talus, observation of the curves above and below the lateral malleoli and the extent of the inversion/eversion of the calcaneus. The observations of the forefoot consist of assessing the bulge in the region of the talo-navicular joint, the congruence of the medial longitudinal arch and the extent of abduction/adduction of the forefoot on the rearfoot [12].

The concurrent validity of the FPI has been investigated fully and reported previously [12]. A more recent study has also demonstrated good internal construct validity and fit of the scoring system to the Rasch model, a useful statistical model of the uni-dimensionality (capacity to measure a single construct) and scale stability (or linearity across a range of values) of a measure [13]. The FPI is suitable for a range of clinical applications and yields high quality linear metric data [13]. The original authors now recommend the use of the six item FPI tool, replacing the eight item version reported previously [14,15].

The FPI has been used in a variety of clinical and research settings. The applications of the FPI include studies of biomechanical risk factors for neuropathic ulceration in diabetes [16], identifying foot type as a basis for screening subjects as inclusion or exclusion criteria in clinical research [17,18], investigating the relationship between foot types and risk factors for sports and training injuries [19-21], investigating whether foot posture is associated with falls in older people [22] and as a means of assessing age-related differences in foot structure [23].

One of the limitations of the FPI is that, to date, there have been no normative data available for comparison and reference. The aim of this study therefore, was to establish normative FPI reference values for use in research and to assist in clinical decision making.

## Methods

### Data acquisition

A search was carried out using online databases (Medline, Embase, PubMed) and internet search engines for studies relating to the use of the FPI. The authors of the studies referencing either the eight or six item FPI were contacted via email with a view to capturing the original data. Original, anonymised datasets were received from nine authors in various formats. Observations from 1648 individual participants were provided, originating from 16 studies undertaken in nine centres. Data collated included centre, age, gender, pathology (where relevant), individual item scores for both the left and right foot (where available), FPI (six-item) total scores for the left and right foot (where FPI eight-item scores were provided, the total FPI six score was derived from the individual item scores), and body mass index (BMI) values, where available.

All data provided for the normative analysis was anonymous and local ethical approval had been given for each of the original studies from the relevant institutional research ethics committees. A summary of the datasets obtained is provided in Table 1.

**Table 1:** Summary of datasets obtained for the analysis.

**Dataset**	**Sample** ** size**	**% male** ** and female**	**Age (mean,** ** SD, range)**	**Pathology**	**BMI**
1	101	M = 31.7%, F = 68.3%	Min = 18 Max = 73 Mean = 42.90 SD = 15.003	Misc local MSK problems (n = 101)	no
2	89	M = 37.1%, F = 62.9%	Min = 18 Max = 52 Mean = 31.34 SD = 9.308	Normal (n = 15), Misc local MSK problems (n = 74)	no
3	428	M = 36.9%, F = 63.1%	Min = 18 Max = 96 Mean = 66.18 SD = 20.707	Normal (n = 428)	yes
4	116	Not provided	Min = 4 Max = 57 Mean = 15.91 SD = 16.481	Normal (n = 104), Neurogenic Cavus (n = 12)	no
5	74	M = 100%	Min = 12 Max = 17 Mean = 14.46 SD = 1.681	Normal (n = 74)	no
6	36	M = 83.3%, F = 16.7%	Min = 43 Max = 77 Mean = 60.19 SD = 9.310	Diabetes (with neuropathy) (n = 36)	no
7	224	M = 38.4% F = 61.6%	Min = 23 Max = 82 Mean = 9.46 SD = 12.340	Misc local MSK problems (n = 224)	yes
8	355	M = 52.7% F = 47.3%	Min = 18 Max = 85 Mean = 40.84 SD = 15.409	Normal (n = 161), Neurogenic Cavus (n = 32), Idiopathic Cavus (n = 162)	yes
9	225	M = 47.6% F = 52.4%	Min = 3 Max = 11 Mean = 7.08 SD = 2.459	Normal (n = 225)	yes

### Statistical analysis

The FPI has undergone testing against the Rasch model to determine its internal construct validity [13]. Ordinal data that fits the Rasch model can be transformed to an interval measurement level using logits as the units of measurement. The logit transformed data, providing it meets the other relevant criteria, can also be analysed using parametric statistics. A table of FPI transformed logit values has been established previously [13], and prior to analysis the total FPI scores for left and right feet were transformed to their equivalent logit values. Descriptive and graphical analyses were used for the main dataset and for each sub group. Comparisons of means were undertaken using Student's *t*-test for unpaired data or a one-way ANOVA with Tukey's post hoc test as appropriate to the number of factors. Relationships were explored using scatter plots and Pearson's correlation coefficient.

Reference ranges were defined using the cut points employed previously for similar studies [24], namely:

(i) Normal: values lying in the range, mean +/- 1 standard deviation (SD)

(ii) Potentially abnormal: values 1 to 2 SDs from the mean

(iii) Pathological: values lying outside 2 SDs from the mean

## Results

### Sample characteristics

The total sample comprised 1,648 participants. There were 717 males, 825 females and 116 participants for whom gender was not specified. The mean age was 42.3 years (SD = 25.1) with a range of 3 to 96 years. BMI data were available for 1,101 participants. 1007 of the participants were normals from the control arms of studies, with the remaining 641 having defined pathologies. Only data from normal adults were included in the main analysis, and the data from the pathological groups is reported separately.

### Normal values

The normal adult sample comprised 619 observations of a single limb from each participant. Data were first tested for normality and this was confirmed both graphically and by the calculation of skewness and kurtosis statistics (skewness = 0.118, kurtosis = -0.096). Left and right side data were compared using Student's *t*-test to identify any side-related systematic difference between observations (left side mean = 1.9 [SD = 2.1], right side mean = 1.9 [SD = 2.0]). This difference was not significant (*t *= -0.21, *p *= 0.983). The mean of the logit scores for the normal sample was 2.4 (SD = 2.3). Logit scores were back transformed into FPI raw scores and normal, potentially abnormal and truly pathological ranges defined. These are presented in Table 2.

**Table 2:** Logit scores and back-transformed FPI-6 raw scores for the normal adult population.

	Pathological	Potentially abnormal	Normal range			Potentially abnormal	Pathological
	< -2 SD	-2 SD	-1 SD	Mean	+1 SD	+2 SD	> +2 SD

FPI logit		-2.2	+0.1	+2.4	+4.7	+7.0	
FPI raw score	< -3	-3	+1	+4	+7	+10	> +10

### Sex differences

Data for male and female participants were explored using descriptive statistics and Student's *t*-test for unpaired data. There was no statistically significant difference between the FPI scores of males and females (2.3 ± 2.4 *versus *2.5 ± 2.3; *t *= -1.44, *p *= 0.149).

### Age-related differences

**Figure 1:**
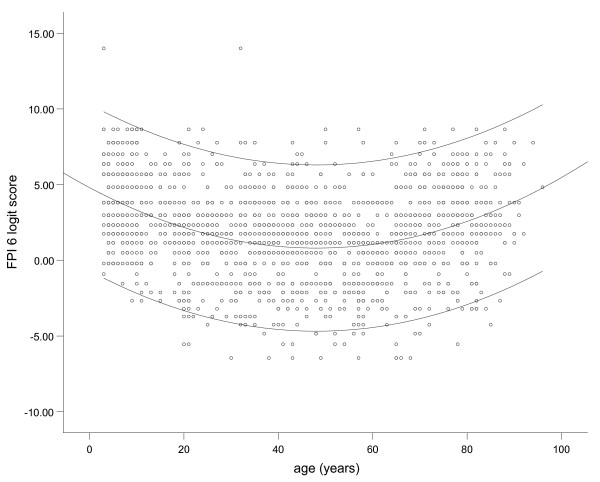
**Scatterplot of FPI scores according to age**.

Normative data were explored for age-related trends and initial scatter plotting suggested that FPI scores may vary with extremes of age (see Figure 1). Within the adult group, those over 60 years appeared to represent a potentially different population, as did a group of minors (n = 388, mean age 8.5 years, range 3 to 17 years) who had been omitted from the analysis outlined in the previous section. The dataset was expanded to include the minors and data were recoded by age group into: normal minors (< 18 years), normal adults (18–59 years) and normal older adults (60 years+).

**Figure 2:**
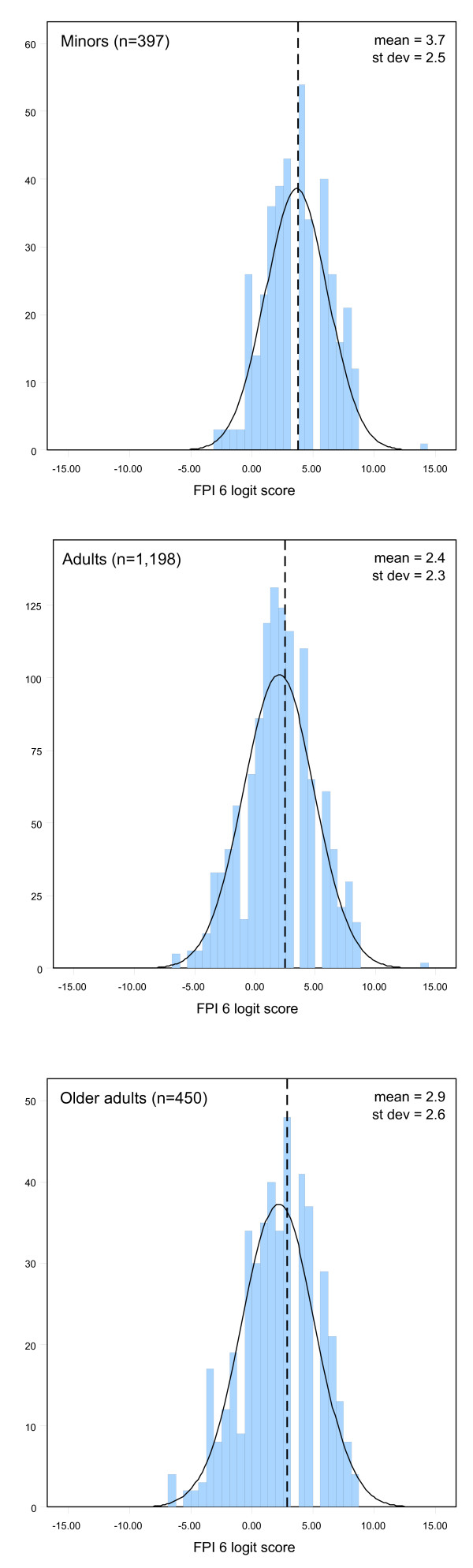
**Histograms of FPI scores for minors, adults and older adults. **Dashed lines represent means.

Graphical output suggested some systematic difference by age group (see Figure 2), with both minors (mean FPI logit score = 3.7, SD = 2.5) and older adults (mean = 2.9, SD = 2.6) showing higher mean scores than the general population. These differences were confirmed with a one way ANOVA (*F *= 51.07, *p *< 0.001). The differences between groups were all confirmed as significant by Tukey's post hoc test (*p *< 0.001). Separate reference ranges have therefore been defined for the minor and older adult groups (Table 3).

**Table 3:** Logit scores and back-transformed FPI-6 raw scores for minors and older adults.

		Pathological	Potentially abnormal	Normal range			Potentially abnormal	Pathological
		< -2 SD	-2 SD	-1 SD	Mean	+1 SD	+2 SD	> +2 SD

Minors (< 18 years)	FPI logit		-1.3	+1.2	+3.7	+6.2	+8.7	
	FPI raw score	< -2	-2	+2	+6	+9	+12	+12
								
Older adults (> 60 years)	FPI logit		-2.3	+0.3	+2.9	+5.4	+8.1	
	FPI raw score	< -3	-3	+1	+5	+8	+11	+12

### Association between FPI and BMI

The dataset was explored for any evidence of a relationship between the FPI and BMI. Scatter-plotting and calculation of Pearson's correlation coefficient identified no relationship between these two variables (*r *= 0.026, *p *= 0.574).

### Differences between pathological groups

**Figure 3:**
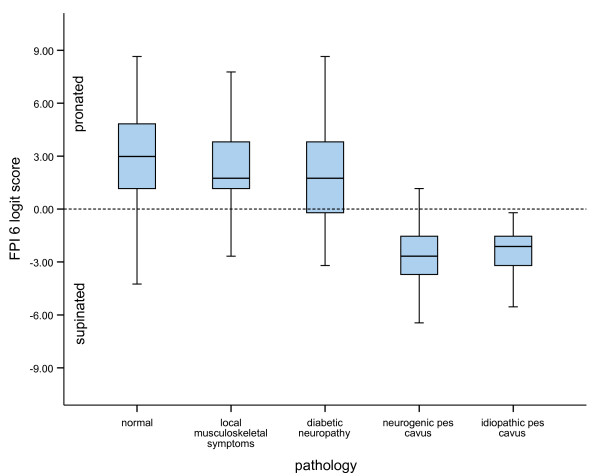
**Boxplots of FPI scores according to presence of pathology**. Error bars are 95% confidence intervals and horizontal lines represent medians.

Finally, the scores from the normal dataset were compared with data from participants with identified pathology who had participated in the relevant studies. Four groups were identified: (i) those with miscellaneous local musculoskeletal symptoms (n = 399); (ii) a group with diabetic neuropathy (n = 36); (iii) a group with neurogenic pes cavus associated with peripheral neuropathy (n = 44), and; (iv) a group with idiopathic pes cavus (n = 162). The means and standard deviations for the miscellaneous musculoskeletal symptoms group and the diabetic neuropathic group were comparable with the normal population, as would be expected for conditions not normally associated with significant structural change. Conversely, the neurogenic cavus and the idiopathic cavus groups were confirmed as representing a clearly pathological population (*F *= 216.981, *p *< 0.001)(see Figure 3).

## Discussion

The FPI is only one of a number of measures of foot posture currently available. Razeghi and Batt [5] discuss the current measures available based on foot morphology and classify them according to four categories: visual assessment, anthropometric values, footprint measures and radiographic appraisal. To date, there are only two foot posture measures – the arch index [7] and the rearfoot angle [10] – for which valid normative data are available. The FPI is the only approach that captures information about standing foot posture in multiple foot segments without a requirement for complex measurement techniques.

The FPI has now been employed in several studies and median FPI raw scores for normal samples have been reported to lie consistently around +5 [19,25]. Other studies have confirmed this tendency towards normal feet as being pronated rather than 'neutral' [20,21]. The current study, employing a large sample indicates that in the normal adult population the mean (back-transformed) FPI score is +4, confirming that a slightly pronated foot posture is the normal position at rest.

Statistically determined reference ranges for postural variations such as standing foot position are inherently wide, so must be used as a general guide only in interpreting FPI scores in a clinical context. It is recognised that clinically, relatively minor variations from the mean may increase risk of mechanically induced pathology, although the strength of these relationships have not been confirmed scientifically and certainly vary for different pathological groups. Except for foot postures falling clearly outside the normal range, the reference ranges alone are probably not adequate for clinical decision making.

There was some evidence of age-related variation in mean foot posture scores and this is in agreement with previous studies. In the recent study by Scott et al [23], a sample of older adults (mean age 80.2 ± S.D. 5.7) had more pronated foot postures than a group of younger adults (mean age 20.9 ± S.D. 2.6). A tendency toward more pronated foot postures in younger children is also well documented. A flatter, more pronated foot has been reported in young children as a consequence of the process of development of the longitudinal arch [8]. The values reported in this study of FPI normative values support the notion of a U-shaped relationship between age and foot posture reported by Staheli et al [8].

While age was found to have an effect on foot posture there was no evidence of any systematic difference between the FPI scores of males (logit mean = 2.3, SD = 2.4) and females (logit mean = 2.5, SD = 2.3). This is again in agreement with the longitudinal arch study by Staheli et al [8] who found minimal differences between male and female foot postures. Although studies have been conducted to analyse foot morphology based on gender [26], studies investigating gender differences in foot posture are limited and our data suggest that gender related differences are small enough to be considered negligible.

The current study found no relationship between BMI and the FPI. Previous studies undertaken using measures such as the footprint angle (FA) and the Chippaux-Smirak index (CSI) have reported lowered longitudinal arches, a broader midfoot area and subsequently flatter feet in people with high BMI values [27]. However, the studies reporting BMI related differences have exclusively used footprint measures, and the postural data may be confounded by the effect of body adiposity on the interpretation of arch height based on these footprint estimates. Indeed, it has been suggested previously that footprint parameters are a measure of "fat feet" rather than "flat feet" [28].

It is known from empirical observation and previous studies that foot posture differences may be encountered in association with underlying disease processes or functional pathology. Comparison of the FPI scores from the normal sample with data from participants known to have identified pathology revealed variations consistent with those predicted by theory. The group with neurogenic pes cavus (mean FPI logit score = -2.78, SD = 2.32) and idiopathic pes cavus (mean = -2.63, SD = 1.25) had FPI scores significantly different from the normal population (mean logit score = +2.4) indicating that the FPI data was sensitive to disease-related postural changes. Data have also been reported elsewhere indicating the sensitivity of the FPI to postural change associated with pathological pes planovalgus (median FPI raw score = +12) [29]. Conversely, the otherwise healthy group with minor musculoskeletal symptoms (mean FPI logit score = 2.23, SD = 2.35) was not systematically different from the normal population (mean FPI logit score = 2.4, SD = 2.3), nor was a sample of patients with diabetes (mean = 2.14, SD = 2.96). There appears therefore to be scope for using FPI scores and associated normative values to help identify groups with structural pathology and to assist in the clinical decision-making process.

There are several limitations to this study that warrant discussion. The most compelling of these is that the data used did not come from a prospectively constructed random sample, such as a general practice or telephone directory derived random sampling frame. Such sampling methods are extremely resource intensive and financially costly whereas the retrospective compilation of a large sample from existing sources covering both normal and pathological subgroups was felt to be a realistic compromise between impact and resource. The dataset was compiled using data from nine centres which raises the possibility of some inconsistency in data collection. One centre had recorded age as a range rather than an integer in years, although sufficient detail was provided to allow classification according to the cut points provided in the analysis. Body mass index was also of less importance to some studies and was not recorded by all centres. However, all incomplete datasets were missing only variables informing the secondary analysis, and for variables of primary importance such as presence or absence of pathology, the dataset was complete.

Observations were derived from either FPI-6 total scores, or through the extraction of the six relevant items from studies using the older eight item version of the FPI. All observers were trained using the official FPI user manual, but it is acknowledged that minor variations in interpretation may have occurred and could not have been controlled for. Conversely, the use of data from multiple centres limits the potential for bias in the total sample and could be considered to enhance the validity of the results.

In summary, this study has provided a set of normative values for FPI scores in a healthy adult population. The data also provides mean and standard deviation values to act as comparators for future studies in a range of potentially pathological groups. Future studies defining FPI ranges of normal and abnormal explicitly according to resulting pathology would supplement this statistical definition and would be helpful to our understanding to the link between foot posture and mechanical 'overuse' type symptoms. The FPI scores did not vary systematically with gender, side of observation or BMI, although did vary at the extremes of age. FPI scores in groups with confirmed structural pathology were systematically different from normal, indicating some sensitivity of the instrument. This now requires further confirmation in specific pathological groups. Further investment in studies to determine definitive reference ranges for children and older adults may help to complete the picture.

## Conclusion

Normative data for the FPI obtained from 619 healthy adults has been presented and compared with grouped data from 1,029 further observations. Based on the analyses presented here, it is concluded that foot posture is influenced by age and presence of pathology, but is not influenced by sex or BMI. The use of age-specific reference ranges provided in this paper will assist in classifying foot type for the purpose of research and clinical decision-making.

## Competing interests

HBM is Editor-in-Chief of the *Journal of Foot and Ankle Research*. It is journal policy that editors are removed from the peer review and editorial decision making processes for papers they have coauthored.

## Authors' contributions

ACR developed the Foot Posture Index, and with HBM, designed the study. YZC coordinated the data capture and statistical analyses. All authors helped draft the manuscript and read and approved the final manuscript.
